# The Duration of Oxygen and Glucose Deprivation (OGD) Determines the Effects of Subsequent Reperfusion on Rat Pheochromocytoma (PC12) Cells and Primary Cortical Neurons

**DOI:** 10.3390/ijms24087106

**Published:** 2023-04-12

**Authors:** Ayesha Singh, Ruoli Chen

**Affiliations:** School of Pharmacy and Bioengineering, Keele University, Staffordshire ST5 5BG, UK; a.singh@keele.ac.uk

**Keywords:** OGD, duration, reperfusion, PC12 cell, rat, neuron, HIF

## Abstract

Reperfusion is the fundamental treatment for ischaemic stroke; however, many ischaemic stroke patients cannot undergo reperfusion treatment. Furthermore, reperfusion can cause ischaemic reperfusion injuries. This study aimed to determine the effects of reperfusion in an in vitro ischaemic stroke model—oxygen and glucose deprivation (OGD) (0.3% O_2_)—with rat pheochromocytoma (PC12) cells and cortical neurons. In PC12 cells, OGD resulted in a time-dependent increase in cytotoxicity and apoptosis, and reduction in MTT activity from 2 h onwards. Reperfusion following shorter periods (4 and 6 h) of OGD recovered apoptotic PC12 cells, whereas after 12 h, OGD increased LDH release. In primary neurons, 6 h OGD led to significant increase in cytotoxicity, reduction in MTT activity and dendritic MAP2 staining. Reperfusion following 6 h OGD increased the cytotoxicity. HIF-1a was stabilised by 4 and 6 h OGD in PC12 cells and 2 h OGD onwards in primary neurons. A panel of hypoxic genes were upregulated by the OGD treatments depending on the duration. In conclusion, the duration of OGD determines the mitochondrial activity, cell viability, HIF-1a stabilization, and hypoxic gene expression in both cell types. Reperfusion following OGD of short duration is neuroprotective, whereas OGD of long duration is cytotoxic.

## 1. Introduction

Ischaemic stroke is the most common type of stroke, affecting 87% of stroke patients. It is caused by the blockage of an artery leading to the brain and supplying a part of the brain region. Cessation of blood flow to a brain region causes reduced blood supply to the region, generating two regions in the affected part of the brain: the ischaemic core and penumbra. In the ischaemic core, due to severe blockage of blood flow, brain cells die mostly via necrosis; in the penumbra, blood flow is reduced, however, brain cells are still alive, and most of them are apoptotic. The cells in the penumbra will be either die or survive, subject to how quickly the blood flow is restored [1]. The ischaemic core region is an area with potentially limited oxygen supply, whereas O_2_ levels in the penumbra range from 1–3% in the brain during ischaemia [2]. Reperfusion via blood clot dissolving or mechanical removal is the only approved treatment for ischaemic stroke at present. However, due to the short time window for the reperfusion treatment and/or contraindications, there are less than 10% ischaemic stroke patients worldwide who have received this treatment [3,4]. Restoration of the vascular supply following ischaemia has often resulted in ischaemia/reperfusion (I/R) injury [5] due to an increase in mitochondrial membrane permeability, resulting in reactive oxygen species generation [2]. Other mechanisms resulting in I/R injury include leucocyte infiltration, platelet activation, complement activation, hyperperfusion, and blood–brain barrier disruption [6].

Over the past 30 years, neuroprotection has received significant attention and shown great promise in experimental studies, but has failed to translate into clinical success [7,8,9]. Several neuroprotective strategies that have failed in the past targeted single molecules (such as glutamate) in the ischaemic cascade, thereby shifting current attention towards targeting the brain’s own evolutionarily conserved endogenous neuroprotective mechanisms. Some of these strategies, such as stem cell therapies, hypothermia, and ischaemic preconditioning, have shown great promise so far [10,11].

The aim of this study was to characterise the neuronal response to oxygen–glucose deprivation (OGD) at different durations (2–24 h) without or with reperfusion (i.e., reintroducing oxygen and glucose). The effects of OGD alone as well as following reperfusion were studied in rat pheochromocytoma (PC12) and primary neurons over time (2, 4, 6, 12, 24 h). Additionally, the expression of hypoxia-inducible factor (HIF) and its downstream genes was studied. HIF is an oxygen sensor that is rapidly activated during hypoxia, and upregulates hundreds of genes that code for various cellular processes [2].

## 2. Results

### 2.1. Responses of PC12 Cells to OGD with or without Reperfusion

**Figure 1:**
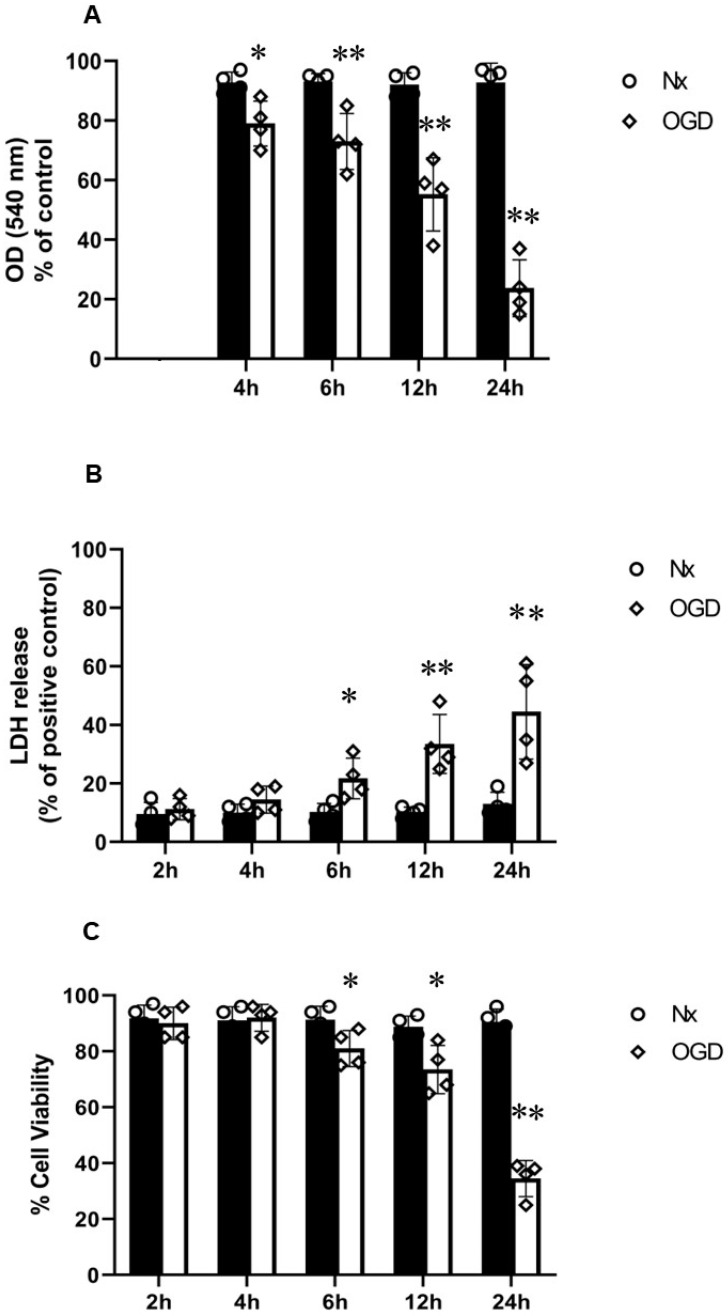
PC12 cells in normoxia (Nx) and subjected to oxygen–glucose deprivation (OGD). (**A**) MTT assay (*n* = 4) showed a significant reduction in MTT activity over time in cells subjected to OGD; (**B**) LDH assay (*n* = 4) showed a significant increase in LDH release in cells subjected to OGD from 6 h OGD onwards; (**C**) trypan blue exclusion assay (*n* = 4) showed a significant reduction in % of live cells from 6 h OGD onwards. Error bars represent ± S.D. * Indicates *p* < 0.05, ** *p* < 0.01, compared to Nx controls.

PC12 cells were subjected to normoxia (Nx) and OGD (0.3% O_2_) for 2, 4, 6, 12, and 24 h (Figure 1). Significant reduction in MTT activity was seen in a time-dependent manner in cells subjected to OGD in comparison to normoxic control (Figure 1A). LDH release significantly increased over time from 6 h of OGD onwards (Figure 1B). Significant reductions in % of live cells (trypan blue exclusion assay) was seen in cells subjected to 6 to 24 h of OGD (Figure 1C).

**Figure 2:**
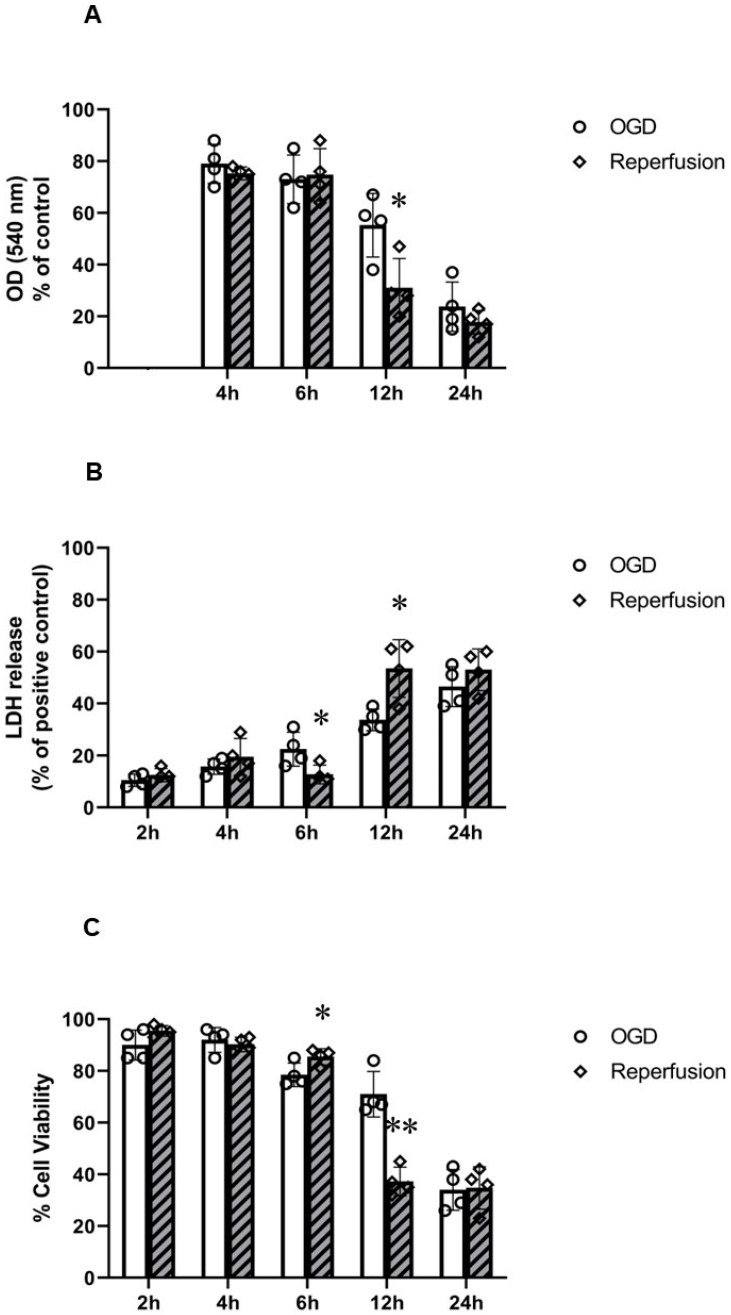
Effects of 24 h reperfusion on PC12 cells following oxygen–glucose deprivation (OGD). (**A**) MTT assay (*n* = 4) showed a significant reduction in MTT activity after 24 h reperfusion in cells previously exposed to 12 h OGD; (**B**) LDH assay (*n* = 4) showed an increase in LDH release in cells pre-exposed to 12 h OGD, but reduced LDH release in cells pre-exposed to 6 h OGD; (**C**) trypan blue assay (*n* = 4) showed a significant reduction in % of live cells in cells pre-exposed to 12 h OGD. Greater viability was seen in cells pre-exposed to 6 h OGD. Error bars represent ± S.D. * Indicates *p* < 0.05, ** *p* < 0.01, for comparison of the treatments of OGD and reperfusion.

After these treatments, cells were subjected to 24 h reperfusion (restoration of oxygen and glucose) (Figure 2). Reperfusion following 2 and 4 h OGD had no significant effects. Reperfusion following 6 h OGD exhibited a reduction in LDH release and an increase in % live cells; meanwhile, reperfusion following 12 h OGD resulted in a further reduction in MTT activity and fraction of live cells and increase in LDH release. The 24 h OGD treatment was damaging to the cells by itself; therefore, no significant changes were seen post reperfusion (Figure 2). These results indicate that whilst reperfusion has a protective effect after exposure to OGD for a short period of time, i.e., 6 h, it has a detrimental effect on cells exposed to OGD for a longer period of time, i.e., 12 h.

**Figure 3:**
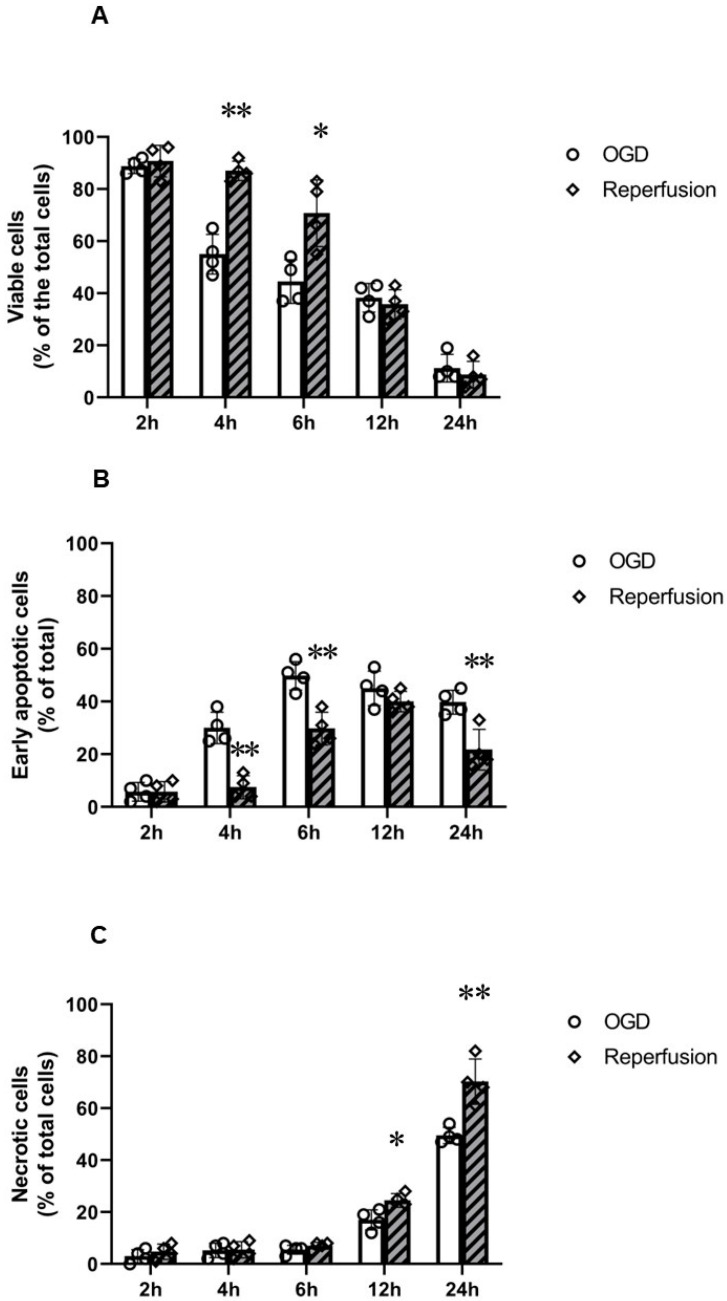
Annexin-V and 7-AAD FACS analysis of PC12 cells following oxygen–glucose deprivation (OGD) before and after 24 h reperfusion. (**A**) Graph of group data (*n* = 4) representing % of live cells (AV^−^/7-AAD^−^); 24 h reperfusion increased % live cells after 4 and 6 h OGD. (**B**) Graph of group data (*n* = 4) representing % of early apoptotic cells (AV^+^/7-AAD^−^); 24 h reperfusion reduced % of early apoptotic cells after 4 and 6 h OGD but increased % of early apoptotic cells after 24 h OGD. (**C**) Graph of group data (*n* = 4) representing % of late apoptotic or necrotic cells (AV^+^/7-AAD^+^); 24 h reperfusion increased % of late apoptotic or necrotic cells after 12 and 24 h OGD. Error bars represent ± S.D. * Indicates *p* < 0.05, ** *p* < 0.01, for comparison of the treatments of OGD and reperfusion.

The effect of OGD alone (without) and following (with) 24 h reperfusion on PC12 cells was thereafter studied by FACS analysis (Annexin-V and 7-AAD double staining) to quantify live (AV^−^/7-AAD^−^), early apoptotic (AV^+^/7-AAD^−^), and necrotic cells (AV^+^/7-AAD^+^) (Figure 3). There was no significant early apoptosis or necrosis by 2 h OGD alone or following reperfusion in comparison to the control. In contrast, 4 h OGD resulted in a significant reduction in % live cells (~55%) and subsequent increase in % apoptotic cells (~30%). Following reperfusion, OGD-induced early apoptosis was significantly reduced and a greater % of live cells (~87%) was seen; no significant necrosis was found following reperfusion. The 6 h OGD treatment resulted in significant reduction in % of live cells (~45%) and subsequent increase in % early apoptotic cells (~50%). Following reperfusion, a reduction in % early apoptotic cells (~30%) and a greater % of live cells (~70%) was seen; no significant necrosis was found following reperfusion. The 12 h OGD treatment resulted in a significant reduction in % of live cells (~38%) and subsequent increase in % early apoptotic cells (~45%) and % necrotic (~17%) cells. Following reperfusion, a reduction in % early apoptotic cells (~32%) and increase in % necrotic cells (~20%) was seen; no significant changes in % of live cells were found following reperfusion. Finally, 24 h OGD resulted in a significant reduction in % live cells (~11%) and increase in % of early apoptotic (~40%) and % necrotic (~54%) cells. Following reperfusion, the % of necrotic (~70%) cells increased further (Figure 3).

**Figure 4:**
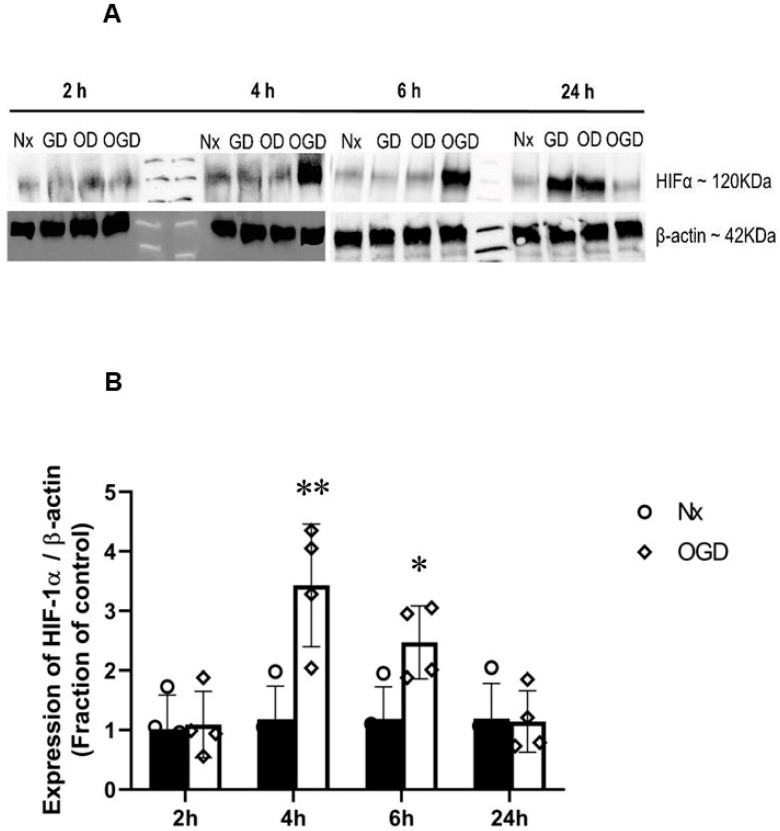
HIF-1α protein stabilisation by oxygen–glucose deprivation (OGD) in PC12 cells. (**A**) Representative Western blots of HIF-1α and corresponding β-actin of cells exposed to 2, 4, 6, and 24 h of OGD. The protein levels were quantified by densitometric analysis using Image J. Values were normalised to β-actin and corresponding Nx control (Figure S1). (**B**) Graph (*n* = 4) showing the normalised HIF-1α level measured after 2, 4, 6, and 24 h OGD. There were significantly higher HIF-1α/β-actin ratios with OGD (4 and 6 h) treatments compared to Nx controls. Error bars represent ± S.D. * Indicates *p* < 0.05, ** *p* < 0.05, against Nx controls.

The effects of OGD on HIF-1α protein expression in PC12 cells were studied. No significant HIF-1α expression was found after 2 h of OGD. HIF-1α was significantly upregulated at 4 h OGD (~4.5-fold); 6 h OGD (~3-fold) significantly upregulated HIF-1α, but at a level significantly lower than 4 h OGD (Figure 4). HIF-1α was not stabilised by 24 h OGD (Figure 4), probably due to significant cell damage induced by the 24 h OGD insult (Figure 3).

**Figure 5:**
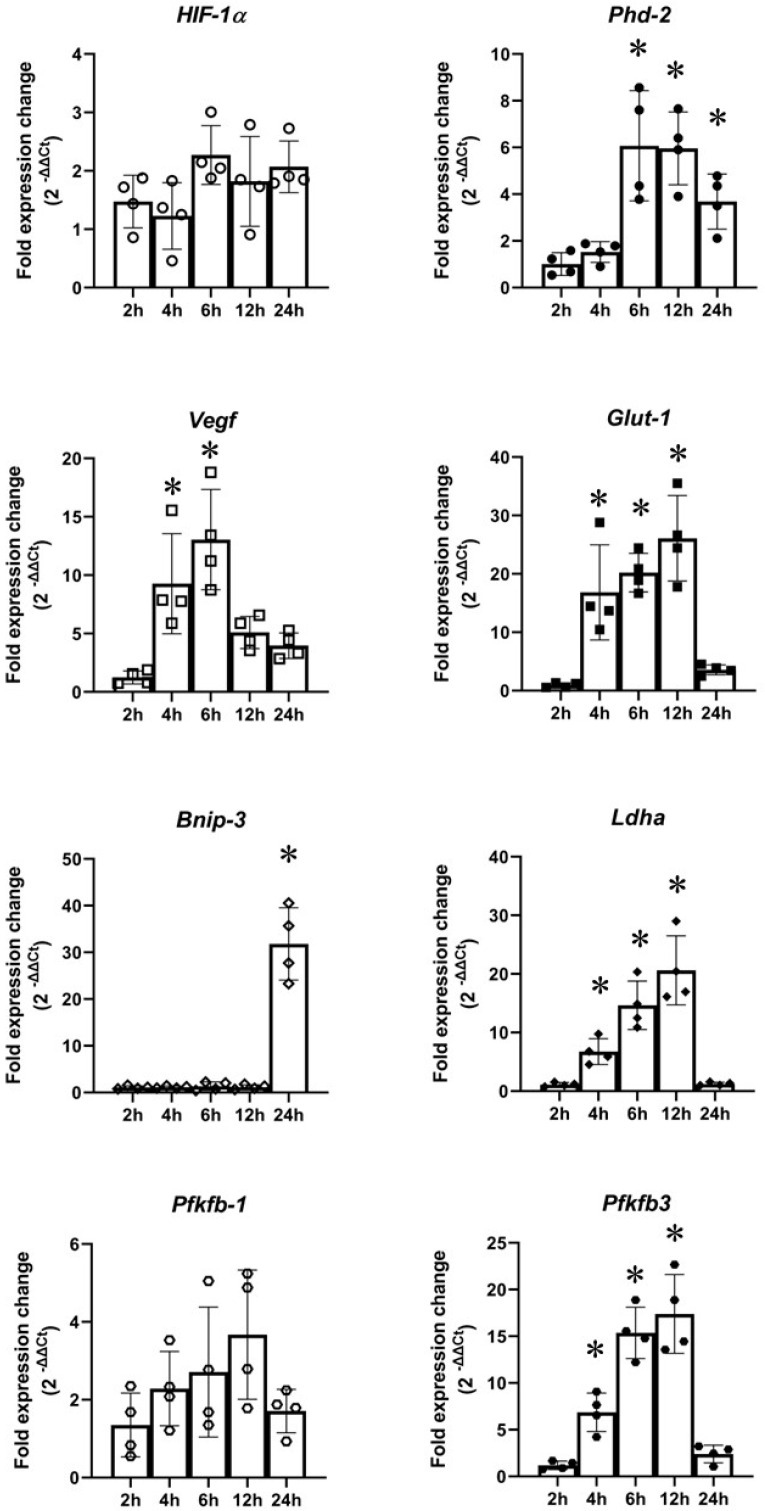
Gene expression in PC12 cells subjected to oxygen–glucose deprivation (OGD). There were no changes in *Hif1α* and *Pfkfb1* expression. There were significant increases in *Phd2* expression from 6 h OGD onwards; significant changes in *Vegf* expression in cells subjected to 4 and 6 h OGD; significant changes in *Glut1* expression in cells exposed to 4, 6, and 12 h OGD; significant changes in *Bnip3* gene expression in cells exposed to 24 h OGD; significant upregulation of *Ldha* in cells subjected to 4, 6, and 12 h OGD; significant upregulation of *Pfkfb3* in cells subjected to 4, 6, and 12 h OGD. The gene expression was measured against housekeeping gene β-*actin.* Error bars represent ± S.D. *n* = 4. * Indicates *p* < 0.05 against normoxia controls.

Gene expression of a panel of hypoxic gene primers was studied using qPCR. As shown in Figure 5, no significant changes in *Hif1α* were seen in cells subjected to OGD at any of the time points. *Phd2* was significant upregulated by OGD at 6 h (~4-fold), 12 h (~6-fold), and 24 h (~4-fold). *Vegf* was significant upregulated by OGD at 4 h (~9-fold) and 6 h (~11-fold). *Glut1* was significantly upregulated by 4 h (~17-fold), 6 h (~21-fold), and 12 h (~25-fold) of OGD. *Bnip3* was significantly upregulated only by 24 h OGD (~32-fold). *Ldha* was significantly upregulated by OGD at 4 h (~8-fold), 6 h (~15-fold), and 12 h (~21-fold). No significant *Pfkfb1* upregulation was seen in PC12 cells subjected to OGD at any of the time points. *Pfkfb3* was significantly upregulated by OGD at 4 h (~7-fold), 6 h (~15-fold), and 12 h (~17-fold).

### 2.2. Responses of Primary Rat Cortical Neurons to Nx and OGD with or without Re-Oxygenation

**Figure 6:**
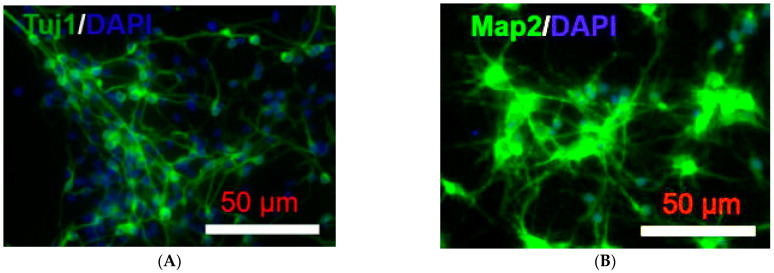
Immunofluorescence images of primary rat cortical neurons. (**A**) Representative double merged (FITC-labelled Tuj1 immunofluorescence (green) and DAPI-stained nuclei) of primary rat cortical neuron culture. The healthy neurons showed various long axons; (**B**) Representative double merged (FITC-labelled MAP2 immunofluorescence (green) and DAPI-stained nuclei) of primary rat cortical neuron culture. The healthy neurons showed numerous dendrites.

Primary rat cortical neurons were isolated from E17,18 embryos, which were represented in Figure 6 and consisted of 65.84% ± 7.23% Tuj1^+^ cells. Healthy neurons consisted of numerous, long axons (Tuj1 staining) and dendrites (MAP2 staining) (Figure 6).

**Figure 7:**
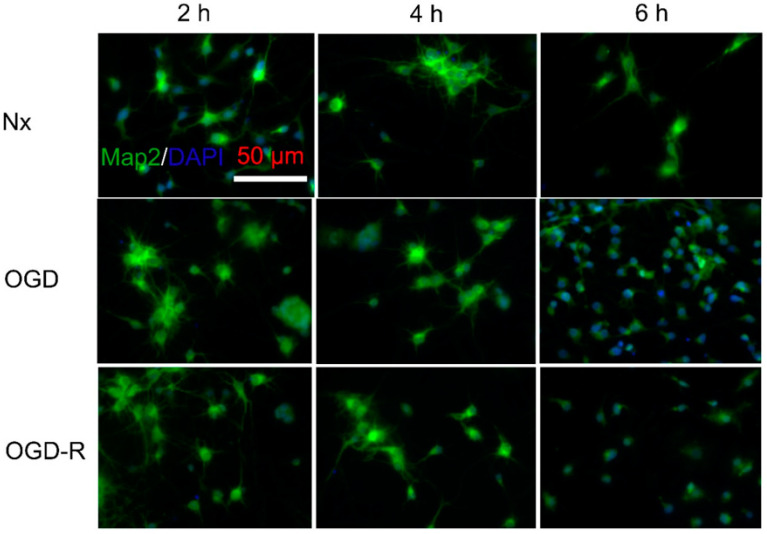
Immunofluorescence images (with MAP2) of primary cortical neurons subjected to oxygen–glucose deprivation (OGD) before and after reperfusion by. Representative double merged (FITC-labelled MAP2 immunofluorescence (green) and DAPI-stained nuclei) revealed control neuron with numerous dendrites; similar neurons were in neurons subjected to 2 and 4 h OGD before and after 24 h reperfusion. Neurons subjected to 6 h OGD, however, displayed neurons without dendrites; and reduced nuclei/microscopic field was found following 24 h reperfusion.

Morphological changes in primary neurons subjected to OGD for 2, 4, and 6 h, before and after reperfusion, were evaluated by the MAP2 immunofluorescence (Figure 7). Healthy cultures in Nx controls consisted of neurons surrounded by numerous MAP2^+^ dendrites (7.6 ± 4.2 dendrites per neuron, with each dendrite measuring 31.4 ± 12.69 mm). OGD (2, 4 h) did not alter the neuron morphology, dendrite length, or dendrite numbers, neither were with 24 h reperfusion. Meanwhile, 6 h OGD resulted in significant changes in neuron morphology compared to the Nx controls at the same time point. There was degradation of dendrites: 1.8 ± 0.6 dendrites per neuron, with each dendrite measuring 5.2 ± 2.4 mm (*p <* 0.05 versus 6 h Nx control). After reperfusion, the dendrites were degrading and there was a loss of MAP2^+^ neurons indicating degeneration of neurons (Figure 7).

**Figure 8:**
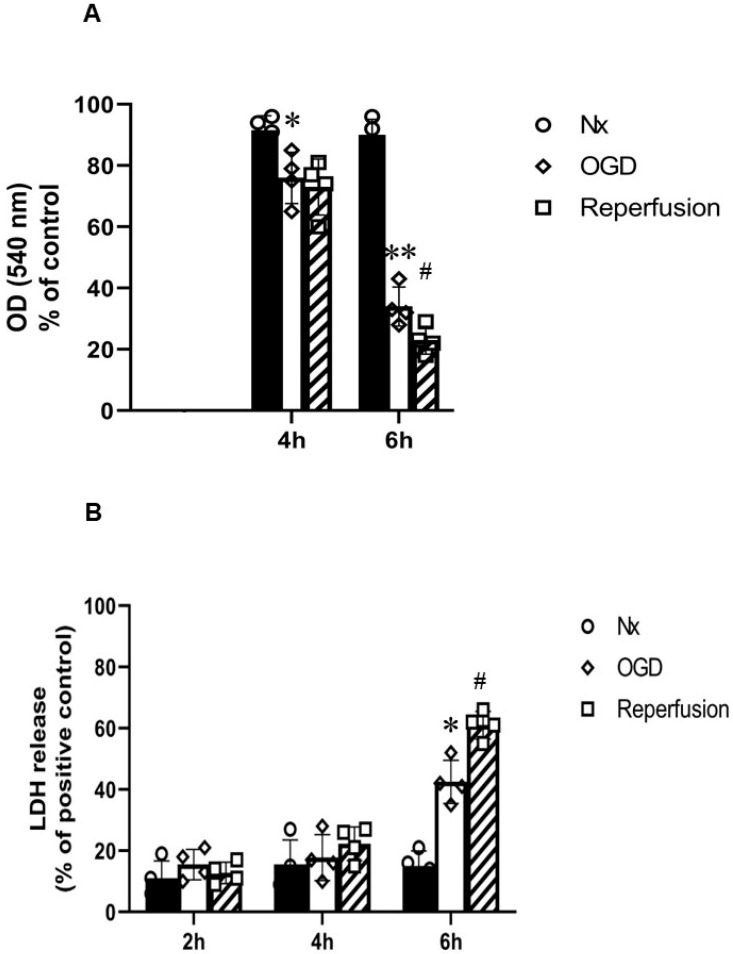
Primary rat cortical neurons in normoxia (Nx) and subjected to oxygen–glucose deprivation (OGD) before and after reperfusion. (**A**) MTT assay (*n* = 4) showed significant reduction in MTT activity over time in primary neurons subjected to 4 and 6 h OGD. A further reduction in MTT activity was found with 24 h reperfusion after 6 h OGD; (**B**) LDH assay (*n* = 4) showed significant increase in LDH release at 6 h OGD, and a further significant increase in LDH release after 24 h reperfusion. Error bars represent ± S.D. * Indicates *p* < 0.05, ** *p* < 0.01, against Nx controls; # indicates *p* < 0.05 for comparison of the treatments of OGD and reperfusion.

MTT assay results revealed that 4 and 6 h of OGD resulted in a significant reduction in MTT activity. A further reduction in MTT activity was seen after 24 h reperfusion in cells exposed to a prior 6 h OGD insult. A significant increase in LDH release was seen by 6 h of OGD insult. A further increase in LDH release was seen after 24 h reperfusion (Figure 8).

**Figure 9:**
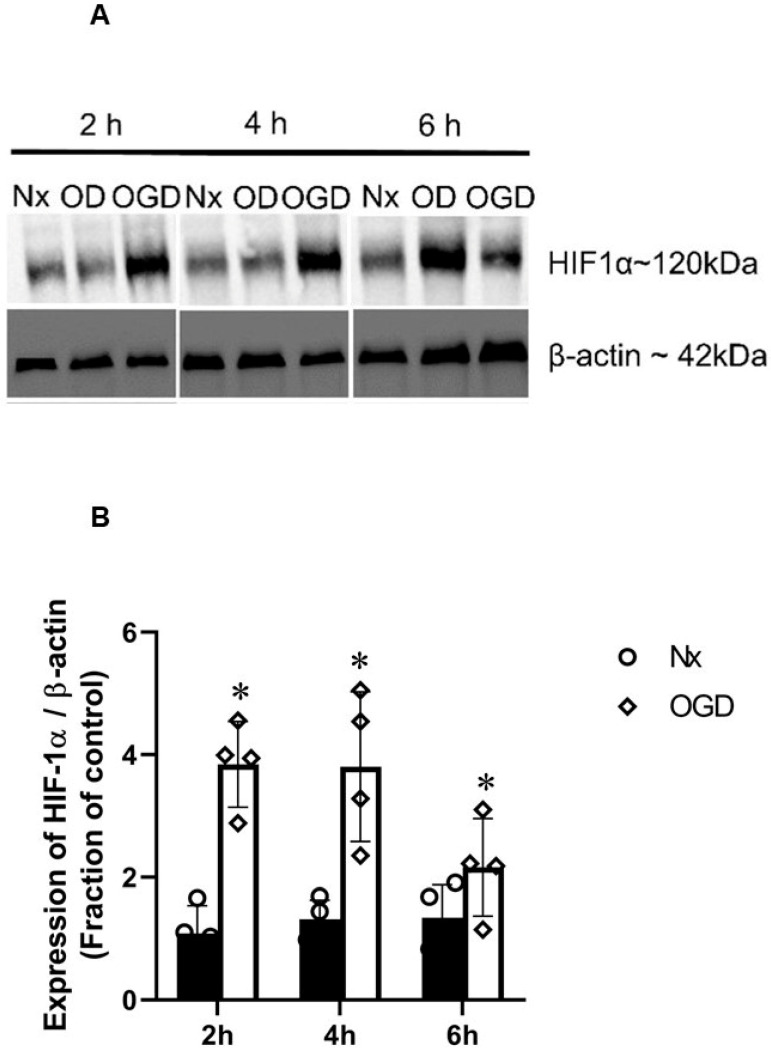
HIF-1α protein stabilisation by oxygen–glucose deprivation (OGD) in primary rat cortical neurons. (**A**) Representative Western blots of HIF-1α and corresponding β-actin of cells exposed to 2, 4, and 6 h of OGD. The protein levels were quantified by densitometric analysis using Image J. Values were normalised to β-actin and corresponding Nx control (Figure S2). (**B**) Graph (*n* = 4) showed the normalised HIF-1α level measured after 2, 4, and 6 h OGD. There was significant higher HIF-1α/β-actin ratios by OGD (2, 4 and 6 h) treatments compared to Nx controls. Error bars represent ± S.D. * Indicates *p* < 0.05 against Nx controls.

The expression of HIF-1α was studied in primary neurons exposed to 2, 4, and 6 h OGD in comparison to normoxia control (Figure 9). Significant HIF-1α stabilisation was seen by 2, 4, and 6 h of OGD.

**Figure 10:**
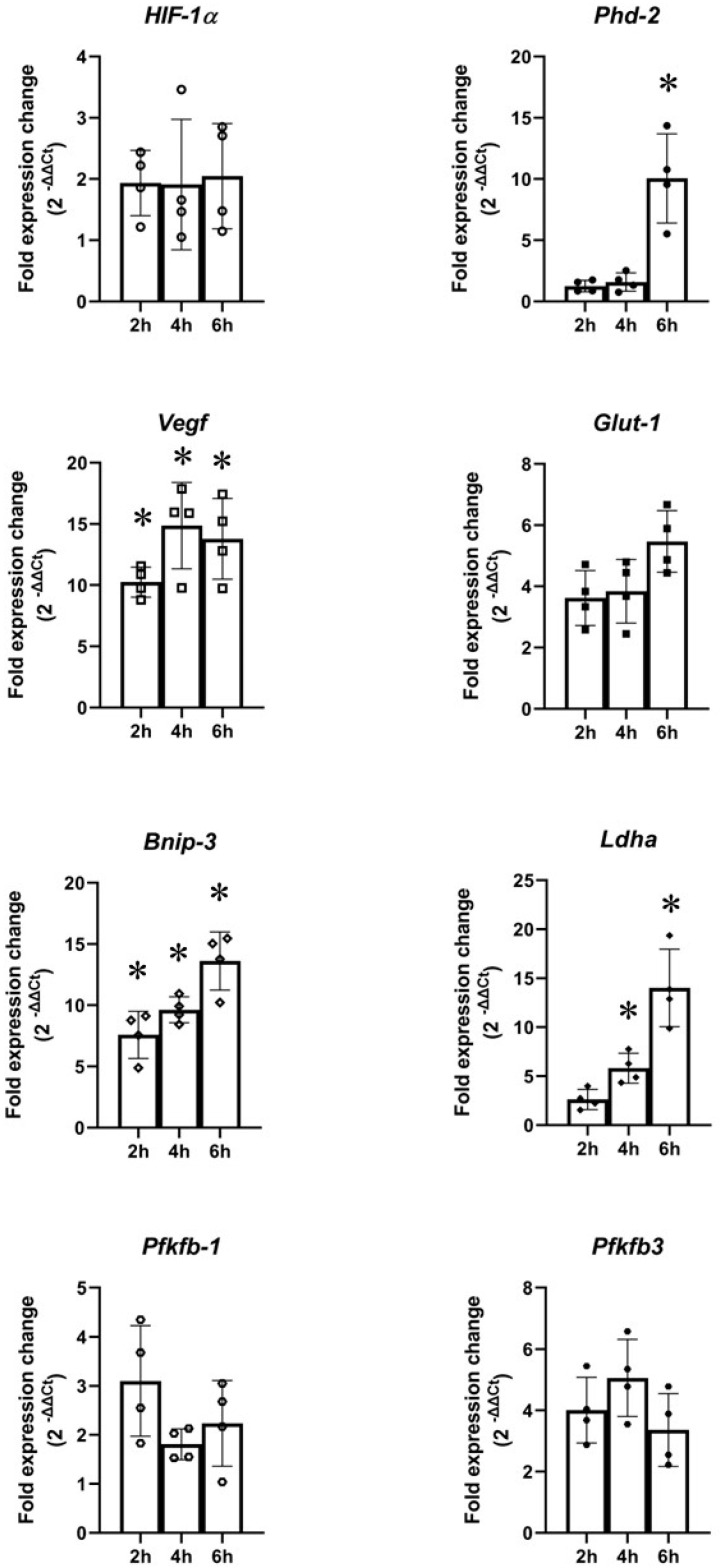
Gene expression in primary rat cortical neurons subjected to oxygen–glucose deprivation (OGD)**.** There were no changes in *Hif1α*, *Glut1, Pfkfb1*, or *Pfkfb3.* There were significant increases in *Phd2* expression by 6 h OGD; significant changes in *Vegf* expression in cells subjected to 2, 4, and 6 h OGD; significant changes in *Bnip3* expression in cells exposed to 2, 4, and 6 h OGD; significant upregulation of *Ldha* in cells subjected to 4 and 6 h. The gene expression was measured against housekeeping gene β-*actin.* Error bars represent ± S.D. *n* = 4. * Indicates *p* < 0.05 against normoxia controls.

Using qRT-PCR, the effects of 2, 4, and 6 h OGD on hypoxia gene expressions in primary rat neurons were investigated. No significant *Hif1α, Glut1, Pfkfb1*, or *Pfkfb3* expression was seen with OGD at any of the time points studied. Significant upregulation of *Phd2* was seen with 6 h OGD (~10-fold). Significant *Vegf* upregulation was seen with 2 (~10-fold), 4 (~15-fold), and 6 (~13-fold) h OGD. Significant *Bnip3* upregulation was seen with 2 (~8-fold), 4 (~10-fold), and 6 (~13-fold) h OGD. *Ldha* was significantly upregulated by 4 (~6-fold) and 6 (~14-fold) h OGD (Figure 10).

## 3. Discussion

This study investigated the effects of OGD (0.3% O_2_) with or without reperfusion in PC12 cells and primary rat cortical neurons. We found that in PC12 cells, there was a significant increase in cytotoxicity from 2 h OGD onwards; however, a longer period (6 h) was required to lead to cytotoxicity in primary neurons. The extent of OGD required for HIF-1α stabilisation and hypoxic gene expression also varied between the cells. In PC12 cells, 4 and 6 h OGD stabilised HIF-1α, whereas in primary neurons, a shorter period (2 h) was sufficient to stabilise HIF-1α. Reperfusion after 6 h OGD in PC12 cells reduced LDH release and increased % cell viability, whereas reperfusion after 12 h OGD further increased LDH release and the percentage of necrotic cells, and reduced % cell viability. In primary neurons, 6 h OGD caused reduced MTT activities and increased LDH release, which were exacerbated following 24 h reperfusion.

PC12 cells isolated from the adrenal medulla of rats have been extensively used in neurobiological studies such as the investigation of signal transduction mechanisms, apoptosis, calcium signalling, Alzheimer’s disease, Parkinson’s disease, and Huntington’s disease [12]. PC12 cells closely resemble neurons with smaller vesicles and quantal size. Undifferentiated PC12 cells express several characteristics of neurons, such as the ability to synthesise, store, and release dopamine and norepinephrine in response to extracellular potassium ion concentration, rise in nicotinic acetyl cholinesterase receptor, and existence of gamma aminobutyric acid (GABA), acetylcholinesterase, and tyrosine hydroxylase [13].

PC12 cells showed significant reductions in MTT activity and increases in LDH release at 2 h OGD onwards, whereas 2 h OGD did not result in any significant primary neuron death in this study. Heravi et al. [14] found that primary neurons were more resistant to alpha-synuclein (Parkinson’s disease) cytotoxicity than PC12 cells. The 6 h OGD treatment was found to be cytotoxic to primary neurons, which worsened following 24 h reperfusion. Meanwhile, in PC 12 cells, 24 h reperfusion showed cytoprotective effects after 6 h OGD but further increased the cytotoxicity after 12 h OGD. The MTT activity at 2 h after OGD cannot be obtained, as there is a 4-h MTT incubation period before the analysis during MTT assay experiments. The metabolised MTT was measured via absorbance using a plate reader after the MTT was added to cells for 4 h to allow the MTT to be taken in and metabolised. Overall, both the neuron-like PC12 cell line and the primary rat cortical neurons displayed similar sensitivities towards in vitro ischaemia and I/R injury.

The oxygen levels of 0.3% were used for OGD experiments in this study. The oxygen level (0.3%) is the lowest possible level in the hypoxia chamber from Ruskinn Technology, and resembles the oxygen level in the ischaemia core region [15]. A significant time-dependent reduction in MTT activity and increase in LDH release was seen from 2 h OGD onwards in PC 12 cells. This is consistent with a study by Li et al. [16], who demonstrated a significant reduction in cell viability over time from 2 h OGD (1% O_2_) onwards. Singh et al. [17] also found a significant reduction in MTT activity from 2 h OGD onwards (0.3% O_2_).

In the trypan blue exclusion assay, there were no significant changes in % live cells in PC12 cells subjected to 2 and 4 h OGD, whereas OGD for 6 h or more resulted in a significant reduction in % live cells. MTT is a reliable indicator of cellular metabolic activity, whereas the LDH assay is representative of membrane depolarisation and measures cell membrane integrity [18]. The trypan blue assay is a dye exclusion assay that relies on the principle that live cells possess an intact cell membrane that excludes the dye, whereas dead cells do not. However, it is important to consider that lethally damaged cells may require several days to lose membrane integrity, the surviving cells continue to proliferate, and some lethally damaged cells may not appear stained with dye as they may undergo early disintegration. The trypan blue assay, therefore, may result in the underestimation of cell death [19].

Our study shows that reperfusion following 12 h OGD in PC 12 cells resulted in further reductions in % live cells and MTT activity, and an increase in LDH release. This is in line with the findings of Singh et al. [17], in which cell viability was studied in PC12 cells subjected to 1–8 h OGD followed by 24 h reperfusion. A further reduction in cell viability was found 24 h after reperfusion in cells subjected to 6 h OGD, and that are therefore considered an ideal model of I/R injury in vitro [17]. Similarly, 6 h OGD followed by 24 h reperfusion reduced cell viability to ~50% and increased LDH release to ~35% [20].

The 2 h OGD insult did not induce apoptosis; however, 4 h OGD resulted in a significant reduction in % live cells (~55%) and subsequent increase in % apoptotic cells (~35%) in PC 12 cells. This is in line with a study by Song et al. [21], in which the authors found a significant increase in % of apoptotic cells from 4 h OGD onwards. Reperfusion following 4 h OGD resulted in a significant reduction in early apoptosis and a greater % of live cells was seen. Li et al. [22] found that apoptotic cells decreased to ~10% of its original level following 4 h OGD with 24 h reperfusion in PC12 cells. On the contrary, 6 h OGD resulted in early apoptosis (~50%) but not necrosis, which worsened following reperfusion (~30% early apoptosis and ~40% of necrotic cells). These results are in line with the LDH and MTT assay results, where reperfusion worsened the outcomes of longer periods of OGD insults (e.g., 12 h). This is in line with a study by Yuan et al. [20], in which 24 h reperfusion following 6 h OGD resulted in ~30% apoptosis and ~50% cell death. Similarly, 24 h OGD resulted in significantly early apoptosis (~40%) and necrosis (~50%), which worsened following reperfusion (~70% necrosis). Sun et al. [23] found that 12 h OGD resulted in ~38% of late apoptotic cells, which further increased to ~50% following reperfusion. Guo et al. [24] also found that 12 h OGD–reperfusion resulted in ~45% of apoptosis.

In this study, neuron morphology was initially assessed using MAP2 and Tuj1 staining. Tuj1 is part of the tubulin family, and are major building blocks for microtubules and structural components of the cytoskeleton. Tuj1 is neuron-specific; it is found in cell bodies, dendrites, axons, and axonal terminals of immature and differentiated neurons. Tuj1 reveals the alteration of the cytoskeleton and progressive loss of neurites upon injury [25]. MAPs are a family of proteins that bind to and stabilise microtubules. MAP2 is a neuron-specific isoform that is involved in the assembly of tubulin into microtubules. MAP2 is primarily expressed in the dendrites of mature neurons, influencing the density of microtubules and the length of dendrites [26]. The MAP2 staining was used subsequently for understanding stress-related changes in neuron structure in our study, as it specifically stained the soma of mature neurons and was less complex. In response to neuronal stress (such as OGD), MAP2 staining reveals the loss of dendritic immunoreactivity, progressing to the complete loss of staining [27].

We found a significant reduction in MTT activity by 4 h OGD in primary rat neurons, which did not change following reperfusion; however, there were no significant changes with the 4 h OGD in LDH release or neuron structure change with MAP2 immunofluorescence. The 6 h OGD treatment resulted in a significant reduction in MTT activity and an increase in LDH release. Similarly, MAP2 staining revealed that 6 h OGD resulted in neurons with reduced numbers of dendrites in comparison with the control (healthy culture consisting of neurons with numerous dendrites). A further reduction in MTT activity and an increase in LDH release was found following 24 h reperfusion. MAP2 staining revealed a reduction in the nuclei/microscopic field consisting of neurons without neurites. Similar results were reported in a study in which 6 h OGD (0% O_2_) resulted in significant neuronal death (~56.7% vs. 19.5% in LDH) and apoptosis (16.5% vs. 2% by TUNEL assay) [28]. Tian et al. [29] also showed a significant increase in LDH leakage (~3-fold vs. control) and reduced cell viability (~50% vs. 100% in control) with 6 h OGD (1% O_2_) in primary rat cortical neurons. In contrast, Bhuiyan et al. [30] showed that 4 h OGD (0% O_2_) followed by 24 h reperfusion resulted in significant cytotoxicity (~60% vs. 10% control in LDH). Another study also showed that 1 h of OGD followed by 24 h of reperfusion resulted in a decrease in viability of ~20%, indicating a moderate degree of cellular stress compared to 3 h OGD, where the viability decreased by more than 50% [31]. Both Bhuiyan et al. [30] and Wappler et al. [31] used EBSS (Earl’s balanced salt solution) for OGD conditions, in contrast to the present and other studies, which used glucose-free Neurobasal medium supplemented with B27. A recent study by Sunwoldt et al. [32] found that B27 protected neurons from cell death during OGD in comparison with neurons incubated in EBSS. Bhuiyan et al. [30] also found that DIV10 rat cortical neurons were more resistant to OGD–reperfusion injury than DIV7 neurons. For this study, mature (DIV10-14) neurons were used, due to which the impact of OGD insult varied from other studies. Factors such as the components of the OGD medium, neuron maturity, and the origin of the neurons account for the differences found in the abovementioned studies [28,29,30,31,32].

HIF is an oxygen-sensing molecule and a key regulator of the genetic response following ischaemia/hypoxia. In PC12 cells, 4 and 6 h OGD stabilised HIF-1α, whereas in primary neurons, significant HIF-1α upregulation commenced earlier, from 2 h OGD onwards. The magnitude of HIF-1α activation depends on the extent of oxygen deprivation; however, glucose also plays a role in the degree of HIF-1α activation [12]. These results indicate that PC12 cells and primary neurons have varying sensitivities to OGD.

In both cell types, no significant change in *Hif1α* gene expression was detected with OGD. This result is consistent with various studies that indicate that HIF-1α levels are mainly regulated at the post-transcriptional level during hypoxia and ischaemia [33]. *Phd2* was significantly upregulated with 6 h OGD in both PC12 cells and primary neurons. In response to HIF protein stabilisation, PHD2 is upregulated as a negative feedback response [34].

*Glut1* was upregulated by OGD (4, 6, 12 h) in PC12 cells. No significant changes in *Glut1* were found in the primary neurons. Studies have reported that GLUT1 is predominantly expressed in astrocytes and endothelial cells in the brain [35]. GLUT-1 upregulation during hypoxia is considered as an adaptive mechanism to increase glucose uptake, allowing cells to maintain or regain ATP levels by increasing the flux through glycolytic pathways during ischaemia [36].

Glycolytic genes such as *Pfkfb1*, *Pfkfb3*, and *Ldha* were studied during OGD in both cells. In primary neurons, *Pfkfb3* was not significantly upregulated by OGD over time. In PC12 cells, varying durations of OGD (4, 6, 12 h) significantly upregulated *Pfkfb3*. PFKFB3 was upregulated in PC12 cells because they are proliferating cancer cells [37], whereas in the brain, PFKFB3 is abundantly expressed in astrocytes but is not typically found in neurons [38]. No significant changes in *Pfkfb1* were seen in PC12 cells or primary neurons, as PFKFB1 is restricted to muscle and liver cells [37]. In contrast, 4 h OGD (~10-fold) and 6 h (~14-fold) OGD upregulated *Ldha* in the primary neurons. Similarly, in PC12 cells, 4 h (~8-fold), 6 h (~15-fold), and 12 h (~21-fold) OGD upregulated *Ldha.*

No upregulation of *Vegf* was seen in PC12 cells at 2 h; however, in the primary neurons, a significant upregulation of *Vegf* was initiated at 2 h and sustained (~15-fold) upregulation found in 4 and 6 h OGD. This is in line with a study showing Vegf mRNA was increased by 4 h ischaemia in neurons [39]. In PC12 cells, a lower level of upregulation in *Vegf* was seen at 4 h (~9-fold) and 6 h (~11-fold) OGD in comparison with primary neurons. VEGF plays an important role in neuroprotection, as it mediates angiogenesis and neurogenesis during ischaemia, resulting in increased blood flow and metabolism [40,41,42].

*Bnip3* levels in PC12 cells were not changed during the shorter period of OGD (from 2 to 12 h), but were upregulated by 24 h OGD. This is consistent with a study where *Bnip3* was upregulated at time points starting at 12 h, with a peak at 72 h in PC12 cells subjected to hypoxia/ischaemia [43]. In contrast, a time-dependent increase in *Bnip3* from 2 h OGD onwards was seen in the primary neurons. *Bnip3* upregulation was reported to be triggered by OGD in primary hippocampal neurons [44]. Studies have shown that *Bnip3* upregulation results in mitochondrial dysfunction, membrane depolarization, MPTP (mitochondrial permeability transition pore) opening, and apoptosis [45,46].

In conclusion, this study demonstrates, using an in vitro stroke model with PC12 cells and rat cortical neurons, that the duration of OGD determines the outcome of subsequent reperfusion. Reperfusion after a short duration of OGD, thus less severity, is neuroprotective, whereas a long duration of OGD is cytotoxic. This study further characterises the time course of HIF-1α stabilisation and its downstream gene expressions following OGD, and shows different patterns of HIF signalling pathway activation in PC12 cells and primary neurons. HIF-1α stabilisation in both cells does not correlate with their viabilities following OGD. This study provides new insights into stroke pathology, where reperfusion is a commonly used treatment for ischaemic stroke, and will lead to the development new therapies by targeting hypoxia signalling pathways.

## 4. Materials and Methods

### 4.1. Materials

Rat adrenal pheochromocytoma (PC12) cells, Dulbecco’s modified Eagle’s medium (DMEM) containing high glucose (4.5 g/L), Dulbecco’s phosphate buffered saline (PBS), fetal bovine serum (FBS), heat-inactivated horse serum (HS), poly-D-lysine (50×), trypsin (50×), 3-(4, 5-dimethylthiazol-2-yl)-2, 5-diphenyltetrazolium bromide (MTT), trypan blue, protease inhibitor cocktail, phenylmethylsulfonyl fluoride (PMSF), Tween-20, Tris, glycine, sodium-dodecyl sulphate (SDS), dithiothreitol (DTT), Triton X-100, paraformaldehyde (PFA), bovine serum albumin (BSA), goat anti-rabbit IgG- FITC, goat anti-mouse IgG- FITC antibody, goat anti-rabbit IgG- FITC antibody were from Sigma-Aldrich (St Louis, MO, USA). Glucose-free Dulbecco’s modified Eagle’s medium, neurobasal medium, glucose-free neurobasal medium, penicillin and streptomycin (10,000 units/mL & 10,000 µg/mL), TrypLE (synthetic trypsin), Glutamax supplement, sodium pyruvate (100 mM), Hank’s balanced salt solution (HBSS), L-glutamine (200 mM), B27 supplement (50×, serum free), Pierce BCA protein assay kit and Pierce ECL Western blotting substrates were from Thermo Fisher Scientific (Loughborough, UK). Laemmli buffer (4×), 4–15% Mini-PROTEAN TGX Precast polyacrylamide gel, skimmed milk, Precision Plus Protein Dual Color Standard were from Bio-Rad (Hertfordshire, UK). Amersham™ Protran^®^ Premium nitrocellulose blotting membranes were from VWR (Leicestershire, UK), RIPA (radio-immuno precipitation assay) buffer (10×) were from New England Biolabs Ltd. (Hertfordshire, UK), Mouse anti- HIF-1α monoclonal antibody was from Novus Biologics (Abington, UK), monoclonal mouse anti-Tuj1 (beta tubulin III) was obtained from Biolegend (San Diego, CA, USA), rabbit polyclonal anti-β-actin antibody and polyclonal rabbit anti-MAP2 (microtubule associated protein 2), were from ABCam (Cambridge, UK), goat polyclonal anti-mouse IgG horseradish peroxidise (HRP) affinity, anti-rabbit IgG HRP affinity were from Dako, Agilent (Santa Clara, CA, USA). Vectashield mounting medium with DAPI was obtained from Vector Laboratories (Burlingame, CA, USA). The Tetro cDNA synthesis kit and SensiFAST^TM^ SYBR Hi-ROX kits were from Bioline Reagents Ltd. (London, UK). The RNeasy plus Mini Kit was from Qiagen (Manchester, UK). The non-radioactive cytotoxicity assay kits were from Promega (Southampton, UK). The Guava cell dispersal reagent, Guava nexin kit, Guava instrument cleaning fluid, Guava Easycheck kit were from Merck Millipore (Burlingon, MA, USA). Plastic materials for cell cultures, including pipettes, T25 cell culture vessel, 96-, 24- and 12-well plates, were from Greiner Bio-One (Gloucestershire, UK).

### 4.2. Cell Culture

#### 4.2.1. PC12 Cells

PC12 cells were cultured in ‘complete’ medium (high-glucose DMEM (containing 4.5 g/L glucose, L-glutamine, and sodium bicarbonate, without pyruvate) supplemented with 5% FBS, 5% HS, and 1% penicillin–streptomycin as described previously [47].

#### 4.2.2. Primary Cortical Rat Neurons Culture

Rat embryos (embryonic day 17–18: E17–18) were removed from a pregnant rat, which was humanely killed under Schedule 1 according to the Animals Scientific Procedures Act of 1986 (UK) with approval from the local ethics committee. The embryonic brains were dissected and the neurons were isolated as described previously [47]. Neurons were then plated onto 5 mg/mL poly-D-lysine pre-coated plates (0.15 × 10^6^ cells per cm^2^) in a standard incubator with a humidified atmosphere containing 5% CO_2_ at 37 °C. Typically, experiments were performed at DIV 9–14.

### 4.3. Cell Treatment Conditions

For normoxic control (Nx), the cells were treated with ‘complete’ medium in a humidified incubator containing 20.9% O_2_ and 5% CO_2_ at 37 °C. For oxygen–glucose deprivation (OGD), the cells were treated with ‘glucose-free’ medium (Glucose-free DMEM containing L-glutamine and sodium bicarbonate, without pyruvate, supplemented with 5% FBS, 5% HS, and 1% penicillin–streptomycin for PC12 cells; glucose-free Neurobasal medium containing 2% B27 serum-free supplements, 2 mM L-glutamine, and 1% penicillin and streptomycin for primary rat neurons) in a purpose-built INVIVO_2_ 400 humidified hypoxia workstation (Ruskinn Technologies, Bridgend, UK) comprising 0.3% O_2_, 5% CO_2_, and 94.7% N_2_ at 37 °C

### 4.4. Assessment of Cell Viability

#### 4.4.1. MTT Assays

Cell mitochondrial activity was evaluated using the standard colorimetric assay as described previously [47]. The activity of mitochondria in control cells (complete media in normoxic conditions) were assigned as 100%, whereas treatment samples were normalised against the control group value.

#### 4.4.2. Lactate Dehydrogenase (LDH) Release Assay

LDH assay was conducted according to manufacturer’s protocol and as described previously [47]. The data are expressed as the mean percent of LDH release from the maximum control.

#### 4.4.3. Trypan Blue Exclusion Assay

Trypan blue exclusion was used to determine the viable cells present in PC12 cell suspensions as described previously [47]. Cell viability is expressed as percentage of viable cells in the total number of cells.

### 4.5. Flow Cytometry Analysis

Apoptosis in PC12 cells was detected with a Guava Nexin Kit containing Annexin V and 7-AAD double stain according to manufacturer’s protocol and as described previously [47]. Data were analysed using Guava analysis software (Merck Millipore, MA, USA) and are expressed as percentage of cells in each quadrant.

### 4.6. Immunofluorescence

Cells were fixed with 4% paraformaldehyde (PFA) for 15 min, and then were permeabilised using 0.1% Triton X-100 in PBS for 15 min, and blocked by incubating with 5% BSA in PBS-T (PBS + 0.1% Triton X-100) for 1 h at room temperature. This was followed by overnight incubation at 4 °C with primary antibody (mouse anti-Tuj1 or rabbit anti-MAP2, 1:500 in 1% BSA in PBS-T). Following three PBS washes, cells were incubated in secondary antibody (goat anti- rabbit IgG-FITC, 1:200 in 1% BSA in PBS-T) for 2 h at room temperature. Coverslips were then washed with PBS and mounted onto slides with Vectashield mounting medium with nuclear stain DAPI (4′, 6-diamidino-2-phenylindole). Images were taken using a Hamamatsu (C4742-95) digital camera attached with Nikon Eclipse 80i fluorescence microscope, and were double merged (consisting of FITC MAP2^+^; DAPI-stained nuclei) with NIS-Element BR 3.22.14 software (Nikon, Tokyo, Japan).

### 4.7. Protein Extraction and Western Immunoblotting

Proteins from the cells after the indicated treatments were extracted as described previously [47]. First, 20 to 40 µg protein was denatured for 5 min in Laemmelli buffer at 95 °C. Samples were electrophoresed and transferred onto a nitrocellulose membrane as described previously [47]. Membranes were blocked with 5% milk powder in 1× PBS-T for 1 h and then incubated overnight at 4 °C with a mouse anti-HIF-1α monoclonal antibody in 1% milk powder of PBS-T buffer. After overnight incubation, the membranes were washed in 1× PBS-T three times for 5 min each, and were incubated for 1 h in the goat polyclonal anti-mouse IgG antibody conjugated with HRP (1:1000) in 1% milk powder of 1× PBS-T. After being washed three times with 1× PBS-T, the membranes were developed using Pierce ECL Western immunoblotting substrates, and imaged with ChemiDoc MP Imaging system (Bio-Rad, CA, USA). Thereafter, the membranes were stripped with mild-stripper buffer and re-probed with a rabbit polyclonal anti β-actin antibody (1:1000) and a subsequent goat polyclonal anti-rabbit IgG antibody conjugated with HRP (1:1000); the image was then taken as describe above. The protein levels were quantified by densitometric analysis using Image J (NIH, Bethesda, MD, USA). Values were normalised to β-actin.

### 4.8. Quantitative Real-Time Polymerase Chain Reaction (qRT-PCR)

RNA was extracted from cells using the RNeasy plus Mini Kit and was converted into cDNA by the Tetro cDNA synthesis kit in accordance with the manufacturer’s protocol, and as described previously [47]. Amplification of 100 ng cDNA template per reaction was performed using the SensiFAST SYBR Hi-ROX kit with a Techne Prime Pro 48 Real-time qPCR machine (ThermoFisher Scientific, Loughborough, UK) as described previously [47]. The primers of a number of genes (*Actin*, *Hif1α*, glucose transporter 1 (*Glut-1*), BCL2/adenovirus E1B 19 kDa protein-interacting protein 3 (*Bnip3*), prolyl hydroxylase 2 (*Phd2*), vascular endothelial growth factor (*Vegf*), 6-phosphofructo-2-kinase/fructose-2,6-biphosphatase 1 (*Pfkfb1*), 6-phosphofructo-2-kinase/fructose-2,6-biphosphatase 3 (*Pfkfb3*), lactate dehydrogenase A (*Ldha*)) used for analysis are described as follows:

*Actin, 5′*-TGCCCTAGACTTCGAGCAAGA-3′ (forward) and 5′-CATGGATGCCACAGGATTCCATAC-3′ (reverse);

*Glut1, 5′*GGTGTGCAGCAGCCTGTGTA-3′ (forward) and 5′-GACGAAC AGCGACACCACAGT-3′ (reverse);

*Hif1α, 5′*-TCAAGTCAGCAACGTGGAAG-3′ (forward) and 5′-TATCGAGGCTGTGTCGACTG-3′ (reverse);

*Vegf, 5′*-TTACTGC TGTACCTCCAC-3′ (forward) and 5′-ACAGGACGGCTTGAAGATA-3′ (reverse);

*Phd2, 5′*-TGCATACGCCACAAGGTACG-3′ (forward) and 5′-GTAGGTGA CGCGGGTACTGC-3′(reverse);

*Bnip3,* TTTAAACACCCGAAGCGCACAG (forward) and GTTGTCAGACGCCTTCCAATGTAGA (reverse);

*Pfkfb1, 5′*-AACCGCAACATGACCTTCCT-3′ (forward) and 5′-CAACACAGAGGCCCAGCTTA-3′ (reverse);

*Pfkfb3, 5′*-CTGTCCAG CAGAGGCAAGAA-3′ (forward) and 5′-CGCGGTCTGGATGGTACTTT-3′ (reverse);

*Ldha, 5′*-AAGGTTATGGCTCCCTTGGC-3′ (forward) and 5′-TAGTGACGTG TGACAGTGCC-3′ (reverse)

*Actin* was used as an internal control to normalise the relative levels of mRNA. Quantification of mRNA expression was performed using the comparative delta Ct method.

### 4.9. Data Analysis

Experiments employing on the 96-well plate reader were performed with 3–8 well replicates. Independent experiments were performed in triplicate. In experiments involving primary neuron cultures, *n* refers to the number of different cultures, each derived from a different rat. The data are expressed as the mean value ± standard deviation (S.D.). One-way ANOVA with Tukey multiple comparison post hoc tests were used to analyse comparisons among multiple groups. GraphPad PRISM 8 for Windows version 10 (GraphPad Software, Inc., San Diego, CA, USA) was used for the analysis.

## Data Availability

The data presented in this study are available on request from the corresponding author.

## Supplementary Materials

The following supporting information can be downloaded at https://www.mdpi.com/article/10.3390/ijms24087106/s1.

## References

[1] Ermine C.M., Bivard A., Parsons M.W., Baron J.-C. (2021). The ischemic penumbra: From concept to reality. Int. J. Stroke.

[2] Chen R., Lai U.H., Zhu L., Singh A., Ahmed M., Forsyth N.R. (2018). Reactive Oxygen Species Formation in the Brain at Different Oxygen Levels: The Role of Hypoxia Inducible Factors. Front. Cell Dev. Biol..

[3] Chia N.H., Leyden J.M., Newbury J., Jannes J., Kleinig T.J. (2016). Determining the Number of Ischemic Strokes Potentially Eligible for Endovascular Thrombectomy: A Population-Based Study. Stroke.

[4] Tsivgoulis G., Katsanos A.H., Sandset E.C., Turc G., Nguyen T.N., Bivard A., Fischer U., Khatri P. (2023). Thrombolysis for acute ischaemic stroke: Current status and future perspectives. Lancet Neurol..

[5] Lee N.T., Selan C., Chia J.S.J., Sturgeon S.A., Wright D.K., Zamani A., Pereira M., Nandurkar H.H., Sashindranath M. (2020). Characterization of a novel model of global forebrain ischaemia–reperfusion injury in mice and comparison with focal ischaemic and haemorrhagic stroke. Sci. Rep..

[6] Kapanova G., Tashenova G., Akhenbekova A., Tokpınar A., Yılmaz S. (2022). Cerebral ischemia reperfusion injury: From public health perspectives to mechanisms. Folia Neuropathol..

[7] Paul S., Candelario-Jalil E. (2021). Emerging neuroprotective strategies for the treatment of ischemic stroke: An overview of clinical and preclinical studies. Exp. Neurol..

[8] Fisher M., Savitz S.I. (2022). Pharmacological brain cytoprotection in acute ischaemic stroke—Renewed hope in the reperfusion era. Nat. Rev. Neurol..

[9] Schneider A.M., Regenhardt R.W., Dmytriw A.A., Patel A.B., Hirsch J.A., Buchan A.M. (2022). Cerebroprotection in the endovascular era: An update. J. Neurol. Neurosurg. Psychiatry.

[10] Papadakis M., Hadley G., Xilouri M., Hoyte L.C., Nagel S., McMenamin M.M., Tsaknakis G., Watt S.M., Drakesmith C.W., Chen R. (2013). Tsc1 (hamartin) confers neuroprotection against ischemia by inducing autophagy. Nat. Med..

[11] Buchan A.M., Pelz D.M. (2022). Neuroprotection in Acute Ischemic Stroke: A Brief Review. Can. J. Neurol. Sci..

[12] Singh A., Chow O., Jenkins S.J., Zhu L.L., Rose E., Astbury K., Chen R.L. (2021). Characterizing ischaemic tolerance in rat pheochromocytoma (PC12) cells and primary rat neurons. Neuroscience.

[13] Chen H., Li H., Cao F., Zhen L., Bai J., Yuan S., Mei Y. (2012). 1,2,3,4,6-penta-O-galloyl-β-D-glucose protects PC12 Cells from MPP+-mediated cell death by inducing heme oxygenase-1 in an ERK- and Akt-dependent manner. J. Huazhong Univ. Sci. Technol..

[14] Heravi M., Dargahi L., Parsafar S., Marvian A.T., Aliakbari F., Morshedi D. (2019). The primary neuronal cells are more resistant than PC12 cells to α-synuclein toxic aggregates. Neurosci. Lett..

[15] Miyamoto O., Auer R.N. (2000). Hypoxia, hyperoxia, ischemia, and brain necrosis. Neurology.

[16] Li J., Zhang S., Liu X., Han D., Xu J., MA Y. (2018). Neuroprotective effects of leonurine against oxygen-glucose deprivation by targeting Cx36/CaMKII in PC12 cells. PLoS ONE.

[17] Singh G., Siddiqui M.A., Khanna V.K., Kashyap M.P., Yadav S., Gupta Y.K., Pant K.K., Pant A.B. (2009). Oxygen Glucose Deprivation Model of Cerebral Stroke in PC-12 Cells: Glucose as a Limiting Factor. Toxicol. Mech. Methods.

[18] Bopp S.K., Lettieri T. (2008). Comparison of four different colorimetric and fluorometric cytotoxicity assays in a zebrafish liver cell line. BMC Pharmacol..

[19] Strober W. (2015). Trypan Blue Exclusion Test of Cell Viability. Curr. Protoc. Immunol..

[20] Yuan J., Zeng L., Sun Y., Wang N., Sun Q., Cheng Z., Wang Y. (2018). SH2B1 protects against OGD/R induced apoptosis in PC12 cells via activation of the JAK2/STAT3 signalling pathway. Mol. Med. Rep..

[21] Song S., Lin F., Zhu P., Wu C., Zhao S., Han Q., Li X. (2017). Extract of Spatholobus suberctus Dunn. ameliorates ischemia-induced injury by targeting miR-494. PLoS ONE.

[22] Li Z., Yang L., Zhen J., Zhao Y., Lu Z. (2018). Nobiletin protects PC12 cells from ERS-induced apoptosis in OGD/R injury via activation of the PI3K/AKT pathway. Exp. Ther. Med..

[23] Sun Y., Zhu W., Zhou S., Wang Z., Chen X., Jia L. (2017). Exploring the model of PC12 apoptosis induced by OGSD/R through in vitro experiments. Oncotarget.

[24] Guo H., Kong S., Chen W., Dai Z., Lin T., Su J., Li S., Xie Q., Su Z., Xu Y. (2014). Apigenin Mediated Protection of OGD-Evoked Neuron-Like Injury in Differentiated PC12 Cells. Neurochem. Res..

[25] Geisert E.E., Frankfurter A. (1989). The neuronal response to injury as visualized by immunostaining of class III beta-tubulin in the rat. Neurosci. Lett..

[26] Soltani M.H., Pichardo R., Song Z., Sangha N., Camacho F., Satyamoorthy K., Sangueza O.P., Setaluri V. (2005). Microtubule-Associated Protein 2, a Marker of Neuronal Differentiation, Induces Mitotic Defects, Inhibits Growth of Melanoma Cells, and Predicts Metastatic Potential of Cutaneous Melanoma. Am. J. Pathol..

[27] Bonde C., Noraberg J., Zimmer J. (2002). Nuclear shrinkage and other markers of neuronal cell death after oxygen–glucose deprivation in rat hippocampal slice cultures. Neurosci. Lett..

[28] Zhang Y., Yang K., Wang T., Li W., Jin X., Liu W. (2017). Nrdp1 Increases Ischemia Induced Primary Rat Cerebral Cortical Neurons and Pheochromocytoma Cells Apoptosis Via Downregulation of HIF-1α Protein. Front. Cell. Neurosci..

[29] Tian T., Zeng J., Zhao G., Zhao W., Gao S., Liu L. (2018). Neuroprotective effects of orientin on oxygen-glucose deprivation/reperfusion-induced cell injury in primary culture of rat cortical neurons. Exp. Biol. Med..

[30] Bhuiyan M.I.H., Kim H.B., Kim S.Y., Cho K.O. (2011). The Neuroprotective Potential of Cyanidin-3-glucoside Fraction Extracted from Mulberry Following Oxygen-glucose Deprivation. Korean J. Physiol. Pharmacol..

[31] Wappler E.A., Institoris A., Dutta S., Katakam P.V., Busija D.W. (2013). Mitochondrial Dynamics Associated with Oxygen-Glucose Deprivation in Rat Primary Neuronal Cultures. PLoS ONE.

[32] Sünwoldt J., Bosche B., Meisel A., Mergenthaler P. (2017). Neuronal Culture Microenvironments Determine Preferences in Bioenergetic Pathway Use. Front. Mol. Neurosci..

[33] Wenger R.H., Kvietiko I., Rolfs A., Gassmann M., Marti H.H. (1997). Hypoxia-inducible factor-1α is regulated at the post-mRNA level. Kidney Int..

[34] Berra E., Roux D., Volmat V., Benizri E., Pouysségur J., Ginouvès A. (2003). HIF prolyl-hydroxylase 2 is the key oxygen sensor setting low steady-state levels of HIF-1α in normoxia. EMBO J..

[35] Winkler E.A., Nishida Y., Sagare A.P., Rege S.V., Bell R.D., Perlmutter D., Sengillo J.D., Hillman S., Kong P., Nelson A.R. (2015). GLUT1 reductions exacerbate Alzheimer’s disease vasculo-neuronal dysfunction and degeneration. Nat. Neurosci..

[36] Yu S., Zhao T., Guo M., Fang H., Ma J., Ding A., Wang F., Chan P., Fan M. (2008). Hypoxic preconditioning up-regulates glucose transport activity and glucose transporter (GLUT1 and GLUT3) gene expression after acute anoxic exposure in the cultured rat hippocampal neurons and astrocytes. Brain Res..

[37] Minchenko A., Leshchinsky I., Opentanova I., Sang N., Srinivas V., Armstead V., Caro J. (2002). Hypoxia-inducible Factor-1-mediated Expression of the 6-Phosphofructo-2-kinase/fructose-2,6-bisphosphatase-3 (PFKFB3) Gene. J. Biol. Chem..

[38] Burmistrova O., Olias-Arjona A., Lapresa R., Jimenez-Blasco D., Eremeeva T., Shishov D., Romanov S., Zakurdaeva K., Almeida A., Fedichev P.O. (2019). Targeting PFKFB3 alleviates cerebral ischemia-reperfusion injury in mice. Sci. Rep..

[39] Zhang Z.G., Chopp M. (2009). Neurorestorative therapies for stroke: Underlying mechanisms and translation to the clinic. Lancet Neurol..

[40] Bernaudin: M., Nedelec A.S., Divoux D., MacKenzie E.T., Petit E., Schumann-Bard P. (2002). Normobaric Hypoxia Induces Tolerance to Focal Permanent Cerebral Ischemia in Association with an Increased Expression of Hypoxia-Inducible Factor-1 and its Target Genes, Erythropoietin and VEGF, in the Adult Mouse Brain. J. Cereb. Blood Flow Metab..

[41] Zhang L., Qu Y., Yang C., Tang J., Zhang X., Mao M., Mu D., Ferriero D. (2009). Signaling pathway involved in hypoxia-inducible factor-1α regulation in hypoxic-ischemic cortical neurons in vitro. Neurosci. Lett..

[42] Patabendige A., Singh A., Jenkins S., Sen J., Chen R. (2021). Astrocyte Activation in Neurovascular Damage and Repair Following Ischaemic Stroke. Int. J. Mol. Sci..

[43] Liu J., Yuan C., Pu L., Wang J. (2017). Nutrient deprivation induces apoptosis of nucleus pulposus cells via activation of the BNIP3/AIF signalling pathway. Mol. Med. Rep..

[44] Zhao S., Chen M., Li S., Zhang M., Li B., Das M., Bean J.C., Kong J., Zhu X., Gao T. (2009). Mitochondrial BNIP3 upregulation precedes endonuclease G translocation in hippocampal neuronal death following oxygen-glucose deprivation. BMC Neurosci..

[45] Chen C., Hu Q., Yan J., Lei J., Qin L., Shi X., Luan L., Yang L., Wang K., Han J. (2007). Multiple effects of 2ME2 and D609 on the cortical expression of HIF-1alpha and apoptotic genes in a middle cerebral artery occlusion-induced focal ischemia rat model. J. Neurochem..

[46] Yeh W.L., Lu D.Y., Lin C.J., Liou H.C., Fu W.M. (2007). Inhibition of Hypoxia-Induced Increase of Blood-Brain Barrier Permeability by YC-1 through the Antagonism of HIF-1α Accumulation and VEGF Expression. Mol. Pharmacol..

[47] Singh A., Wilson J.W., Schofiled C.J., Chen R.L. (2020). Hypoxia-inducible factor (HIF) prolyl hydroxylase inhibitors induce autophagy and have a protective effect in an in-vitro ischaemia.model. Nat. Sci. Rep..

