# Supplementary material for: The Duration of Oxygen and Glucose Deprivation (OGD) Determines the Effects of Subsequent Reperfusion on Rat Pheochromocytoma (PC12) Cells and Primary Cortical Neurons
Source: Int J Mol Sci. 2023 Apr 12;24(8):7106. doi: 10.3390/ijms24087106 (PMC10138834; doi:10.3390/ijms24087106)
Supplement: Supplementary file 1 [file ijms-24-07106-s001.zip › ijms-2321282-supplementary.pdf]

Supplementary data

The Duration of Oxygen and Glucose Deprivation (OGD) Determines the Effects of Subsequent Reperfusion on Rat Pheochromocytoma (PC12) Cells and Primary Cortical Neurons

Ayesha Singh and Ruoli Chen *

School of Pharmacy and Bioengineering, Keele University, Staffordshire ST5 5BG, UK
* Correspondence: r.chen@keele.ac.uk

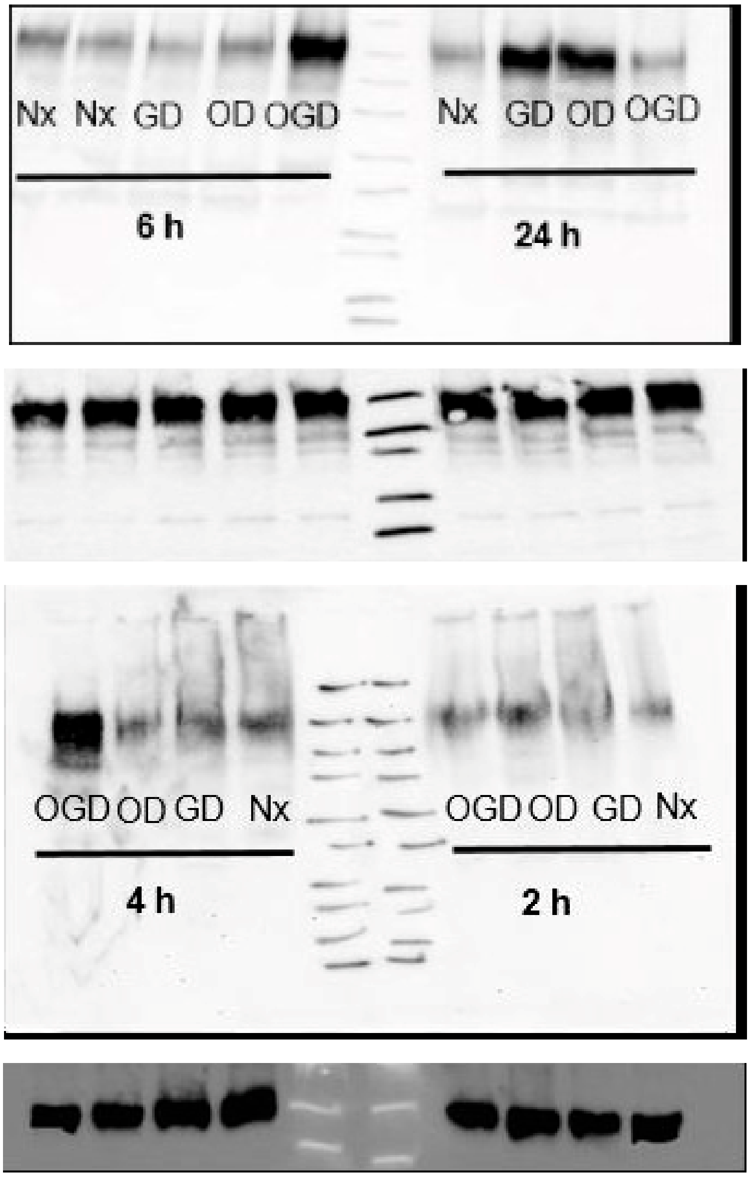

Figure S1. HIF-1α protein stabilisation by oxygen-glucose deprivation (OGD) in PC12 cells. Representative western blots of HIF-1α and corresponding β-actin of cells exposed to 2, 4, 6 and 24 h of OGD. The protein levels were quantified by densitometric analysis using Image J. Values were normalized to β-actin and corresponding Nx control.

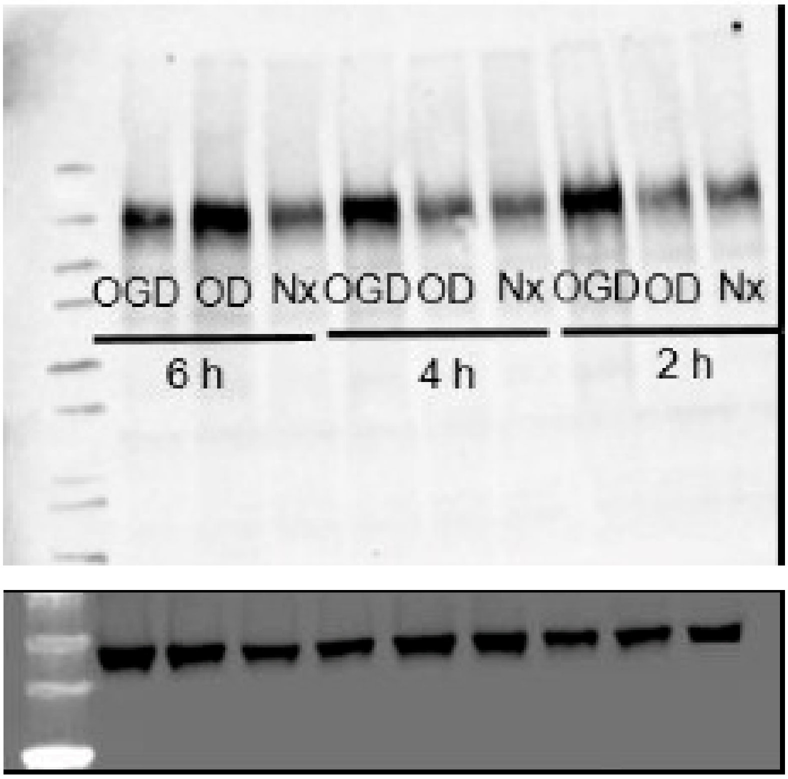

Figure S2. HIF-1α protein stabilisation by oxygen-glucose deprivation (OGD) in primary rat cortical neurons. Representative western blots of HIF-1α and corresponding β-actin of cells exposed to 2, 4, and 6 h of OGD. The protein levels were quantified by densitometric analysis using Image J. Values were normalized to β-actin and corresponding Nx control.
